# Efficacy and safety of early cholecystectomy for comorbid acute cholecystitis and acute cholangitis: Retrospective cohort study

**DOI:** 10.1016/j.amsu.2018.10.031

**Published:** 2018-11-01

**Authors:** Tomoyuki Abe, Hironobu Amano, Keiji Hanada, Tomoaki Bekki, Tomoyuki Minami, Shuji Yonehara, Toshio Noriyuki, Masahiro Nakahara

**Affiliations:** a Departments of Surgery, Onomichi General Hospital, Onomichi, Hiroshima, Japan; b Departments of Gastroenterology, Onomichi General Hospital, Onomichi, Hiroshima, Japan; c Departments of Pathology, Onomichi General Hospital, Onomichi, Hiroshima, Japan; d Department of Gastroenterological and Transplant Surgery, Graduate School of Biomedical and Health Sciences, Hiroshima University, Hiroshima, Japan

**Keywords:** Acute cholangitis, Acute cholecystitis, Cholecystectomy, 2013 Tokyo guideline

## Abstract

**Background:**

This study investigated the optimal timing and usefulness of early cholecystectomy for acute cholecystitis in patients with comorbid acute cholangitis.

**Materials and methods:**

In 2011–2016, 252 patients who underwent early cholecystectomy for acute cholecystitis and 7 who underwent delayed cholecystectomy were enrolled and compared. Patients with comorbid acute cholangitis were then divided into those who underwent urgent cholecystectomy (within 72 h after symptom onset), semi-urgent cholecystectomy (3–14 days after symptom onset), or delayed cholecystectomy (3 months after symptom onset).

**Results:**

There were no significant intergroup differences in postoperative complication rate (*p* = 0.561), operation time (*p* = 0.496), or intraoperative blood loss (*p* = 0.151) between those with and those without acute cholangitis. Postoperative stays were significantly longer in the comorbid acute cholangitis group (*p* = 0.004). In the patients with acute cholangitis, the urgent cholecystectomy, semi-urgent, and delayed cholecystectomy groups had comparable intra- and postoperative outcomes.

**Conclusion:**

Early cholecystectomy within 14 days after symptom onset was safely performed for patients with concomitant acute cholecystitis and acute cholangitis after the successful treatment of acute cholangitis.

## Highlights

• Feasibility and safety of early cholecystectomy for concomitant acute cholecystitis with acute cholangitis within 14 days after symptom onset.
• Upfront treatment for acute cholangitis is essential for achieving low postoperative mortality in case of concomitant acute cholecystitis and acute cholangitis.

## 1 Introduction

Since the revision of the 2013 Tokyo guideline (TG13), early cholecystectomy within 72 h after symptom onset has been recommended for grade 1 (G1) and grade 2 (G2) acute cholecystitis [1]. In the past, acute cholecystitis was considered a contraindication for laparoscopic cholecystectomy (LC); however, due to the widespread use of LC and stylized laparoscopic procedures, it is now considered an indication for LC. Previous studies demonstrated that LC was superior to open procedures for patients with acute cholecystitis [[2], [3], [4]]. Early cholecystectomy boasts several benefits over late cholecystectomy including lower medical costs, fewer postoperative complications, and sooner recovery [[5], [6], [7], [8]]. However, despite the widespread use of LC, intraoperative biliary injury remains the major complication resulting from misdirection. Recent data show that the gallbladder wall in the presence of acute edematous and fibrotic changes induced by acute inflammation, but not chronic fibrotic changes, is not obscured in the optimal view and dissection of Calot's triangle [9]. Based on this theory, LC is now recommended as the first-choice treatment for acute cholecystitis.

Clinicians encounter patients with concomitant acute cholangitis and acute cholecystitis (considered concomitant biliary infections), but surgical intervention timing differs among institutions. The appropriate interval between the treatment of acute cholangitis and surgical intervention for acute cholecystitis remains to be elucidated. According to the 2007 Tokyo guideline, elective LC after the successful treatment of acute choledocholithiasis and cholangitis is recommended in stable patients [10], whereas the TG13 fails to describe the optimal timing of surgical interventions. A few reports evaluated the optimal surgical timing instead of the high recurrence rate of biliary symptoms after the treatment of choledocholithiasis [11,12].

Acute cholangitis, one of the most common acute abdominal disorders, frequently features septic shock and organ disorders due to cholangiovenous reflux and requires urgent biliary drainage or antibiotic therapy [13]. In cases of choledocholithiasis, cholecystectomy is subsequently performed to ensure the successful treatment of acute cholangitis and prevent its recurrence. Previous reports demonstrated that the low recurrence rate of acute cholangitis is around 3% during a waiting period of 6–12 weeks for subsequent cholecystectomy [14]. In contrast, a high biliary complication rate of 20% was noted after a median waiting time of 7 weeks, and patients who developed recurrence had worse postoperative outcomes than those who did not [15].

Therefore, the objective of this study is to validate the efficacy and safety of early cholecystectomy in patients with concomitant biliary infections after the successful treatment of acute cholangitis.

## 2 Material and methods

### 2.1 Patients

Between May 2011 and December 2016, 252 consecutive patients who underwent urgent cholecystectomy and 7 patients who underwent delayed cholecystectomy were enrolled in this study. Patients with grade 3 (G3) acute cholangitis were excluded from this study. The exclusion criteria for acute cholangitis were: (1) association with acute pancreatitis; and (2) detected pancreatobiliary malignancies. Acute cholecystitis and acute cholangitis were diagnosed and severity was graded according to the TG13 by analysis of the patient's symptoms, laboratory data, and abdominal ultrasonography and computed tomography (CT) findings. Emergency magnetic resonance cholangiopancreatography (MRCP) was used to observe the anatomy of the extrahepatic bile duct and prevent intraoperative injury to the main bile duct or detect an accessory hepatic duct. This work has been reported in line with the Strengthening the Reporting of Cohort Studies in Surgery criteria [16].

### 2.2 Surgical procedures and perioperative management

LC using the widely accepted four-port procedure was the first choice. The decision to convert from LC to open cholecystectomy was made by the instructor surgeon. Perioperative management was performed as described elsewhere [17,18]. In patients with acute cholangitis, emergency endoscopic retrograde cholangiopancreatography (ERCP) was performed; cases of obstructive jaundice and severe acute cholangitis rather than acute cholecystitis required investigation to determine the laboratory data and general condition recovery.

### 2.3 Treatment strategy for acute cholangitis

Most of our patients with acute cholangitis underwent ERCP. An emergency biliary drainage tube was inserted in most cases, and an endoscopic nasobiliary drainage (ENBD) or endoscopic retrobiliary drainage tube was inserted to provide the drainage. Patients with mild acute cholangitis and no common bile duct stones were treated with antibiotics. After the jaundice and liver dysfunction resolved, early cholecystectomy was performed. Early cholecystectomy was defined as that performed within 14 days of symptom onset. Urgent cholecystectomy was that performed within 72 h from symptom onset, semi-urgent was 3–14 days, and delayed cholecystectomy was 3 months after symptom onset.

### 2.4 Morbidity and complications

The definition of complications was made as described by Clavien et al. In our study, postoperative complications were defined as those that were grade IIIa or greater [19].

### 2.5 Statistical analysis

Continuous variables are presented as median and range, while categorical variables are presented as number (percentage). Continuous variables were compared using the Mann-Whitney *U* test, while categorical variables were compared using Fisher's exact test. The data were then analyzed using single-factor analysis of variance. Values of *p* < 0.05 were considered statistically significant. The statistical analyses were performed using IBM Statistical Package for Social Sciences for Windows version 22.0 (IBM Corp., Armonk, NY, USA).

## 3 Results

A total of 252 patients (163 male, 89 female; age range, 9–99 years) following early cholecystectomy for clinically documented acute cholecystitis according to the TG13 were included in the present study. Hypertension was the most common pre-existing condition (116 patients; 46.0%), followed by type 2 diabetes mellitus (65 patients; 25.8%) and cardiovascular disease (47 patients; 18.7%). Postoperative complications were detected in 31 patients (12.3%); there was 1 case of in-hospital death.

### 3.1 Comparison of clinical variables between patients with concomitant biliary infections (comorbid acute cholecystitis and acute cholangitis) and patients without acute cholangitis

The cohort was subdivided into two groups as follows: patients with concomitant biliary infections (n = 101; 40.1%) and those without acute cholangitis (n = 151; 59.9%). The clinical data of these two groups are shown in Table 1. Older mean age (*p* = 0.001) and a higher incidence of an American Society of Anesthesiologists Physical Status score ≥ 3 (*p* = 0.032) were seen in the concomitant biliary infections group than in the non-acute cholangitis group. Laboratory data at the time of admission revealed worse platelet count (*p* = 0.013), total bilirubin (*p* < 0.001), aspartate transaminase (*p* < 0.001), albumin (*p* = 0.001), and alanine transaminase (*p* < 0.001) levels in the concomitant biliary infections group than in the non-acute cholangitis group. CT findings of acute cholecystitis were comparable between the two groups. Comorbidities of cardiovascular and chronic kidney disease were significantly more common in the concomitant biliary infections group (*p* = 0.048 and *p* = 0.049, respectively). The proportions of G2 acute cholecystitis were similar in the two groups (*p* = 0.367).

**Table 1:** Patient characteristics in the concomitant biliary infections and non-acute cholangitis groups.

	All patients (n = 252)	Concomitant biliary infections (n = 101)	Non-acute cholangitis (n = 151)	*P* value
**Sex, M/F**	163/89	56/45	107/44	**.015**
**Median age, y (range)**	74 (9–99)	77 (26–95)	71 (9–99)	**.001**
BMI, kg/m^2^ (range)	24 (14.6–37.3)	23 (15.8–35.4)	24 (14.6–37.3)	.101
**ASA-PS** ≥ **3 (%)**	46 (18.3%)	25 (24.8%)	21 (13.9%)	**.032**
WBC, × 10^3^/L (range)	12630 (2000–30700)	11490 (2000–30700)	13200 (3602–28600)	.317
Platelet count, × 104/μL (range)	21 (1.5–49.7)	20 (1.9–49.7)	23 (1.5–48.1)	.013
**T-Bil, mg/dL (range)**	1.2 (0.2–9.4)	2.0 (0.4–9.4)	0.9 (0.2–7.7)	**<.001**
**AST, IU/L (range)**	34 (12–876)	131 (13–876)	24 (12–515)	**<.001**
**ALT, IU/L (range)**	37 (7–851)	123 (8–851)	23 (7–495)	**<.001**
**Albumin, g/dL (range)**	3.6 (1.5–6.2)	3.4 (1.9–6.2)	3.7 (1.5–5.3)	**.001**
CRP, mg/dL (range)	7 (0.0–45)	8 (0.0–32)	6 (0.0–45)	.609
Grade 2 acute cholecystitis	113 (44.8%)	49 (48.5%)	64 (42.3%)	.367
Computed tomography findings				
Wall thickness	214 (84.9%)	85 (84.2%)	129 (85.4%)	.858
Gallstones	183 (72.6%)	73 (72.3%)	110 (66.9%)	1.000
Enhanced surrounding tissue	182 (72.2%)	70 (69.3%)	112 (74.2%)	.474
Effusion around gallbladder	54 (21.4%)	22 (21.8%)	32 (21.2%)	1.000
Liver abscess	13 (5.2%)	6 (5.9%)	7 (4.6%)	.773
Ascites	29 (11.5%)	9 (8.9%)	20 (13.2%)	.321
Diabetes mellitus, n (%)	65 (25.8%)	29 (28.7%)	36 (23.8%)	.463
Hypertension, n (%)	116 (46.0%)	49 (48.5%)	67 (44.4%)	.522
Pulmonary disease, n (%)	27 (9.5%)	12 (11.9%)	15 (9.9%)	.680
**Cardiovascular disease, n (%)**	47 (18.7%)	25 (24.8%)	22 (14.6%)	**.048**
**Chronic kidney disease, n (%)**	19 (7.5%)	12 (11.9%)	7 (4.6%)	**.049**

Variables in bold are statistically significant *(p* < 0.05). Continuous variables are expressed as median (range). Qualitative variables are expressed as number (%). ASA, American Society of Anesthesiologists; AST, aspartate transaminase; ALT, alanine transaminase; BMI, body mass index; CRP, C-reactive protein; F, female; M, male; PS, physical status; WBC, white blood cell.

### 3.2 Comparison of perioperative outcomes between patients with concomitant biliary infections and those without acute cholangitis

Intra- and postoperative outcomes are presented in Table 2. The postoperative hospital stay was significantly prolonged in the concomitant biliary infections group (9 days vs 7 days, *p* = 0.004). Operative time and postoperative complication rates were comparable between the two groups (*p* = 0.496 and *p* = 0.561, respectively). Time to operation from symptom onset was longer in the concomitant biliary infections group than in the non-acute cholangitis group (*p* = 0.007). There were no cases of intraoperative biliary injury in this cohort.

**Table 2:** Perioperative patient outcomes in the concomitant biliary infections and non-acute cholangitis groups.

	All patients (n = 252)	Concomitant biliary infections (n = 101)	Non-acute cholangitis (n = 151)	*P* value
Operative procedure				.515
LC (%)	203 (80.1%)	79 (78.2%)	124 (82.1%)	
OC (%)	49 (19.9%)	22 (21.8%)	27 (17.9%)	
Operation time, min	144 (63–269)	142 (71–253)	146 (63–269)	.496
Intraoperative blood loss, mL	30 (0–1337)	40 (0–1200)	20 (0–1337)	.151
Blood transfusion, n (%)	9 (3.6%)	6 (5.9%)	3 (2.0%)	.163
Postoperative complications ≥ CD IIIa, n (%)	31 (12.3%)	14 (13.9%)	17 (11.3%)	.561
**Postoperative stay (days), n (range)**	8 (2–86)	9 (4–86)	7 (2–78)	**.004**
**Operation within 72 h after symptom onset, n (%)**	163 (64.7%)	55 (54.5%)	108 (71.5%)	**.007**

Variables in bold are statistically significant (*p* < 0.05). Continuous variables are expressed as median (range). CD, Clavien-Dindo classification; LC, laparoscopic cholecystectomy; OC, open cholecystectomy.

### 3.3 Evaluation of optimal surgical timing in patients with acute cholangitis

The 108 patients with comorbid acute cholangitis were subdivided into three groups: urgent operation, in which cholecystectomy was performed within 72 h after symptom onset (n = 57); semi-urgent operation, in which it was performed 3–14 days after symptom onset (n = 44); and delayed operation, in which it was performed 3 months after symptom onset (n = 7). The clinical data of the three groups are shown in Table 3. There were significant differences in mean age (urgent vs semi-urgent vs delayed groups of 80 yr vs 73 yr vs 79 yr, *p* = 0.020), Murphy's sign presence (64.9% vs 50.0% vs 14.3%, *p* = 0.022), acute cholecystitis severity grading proportion (G1/2/3; 32/18/7 vs 10/31/3 vs 6/1/0, *p* = 0.001). There were no cases of G3 acute cholecystitis in the delayed operation group. Preoperative inflammation was significantly more common in the urgent and semi-urgent groups than in the delayed group (white blood cell count and C-reactive protein level, *p* = 0.003 and *p* = 0.002, respectively). Preoperative CT findings were significantly different in cases of abscesses around the gallbladder (0 vs 6 vs 0, *p* = 0.010) and gallbladder stones (39 vs 34 vs 1, *p* = 0.004).

**Table 3:** Patient characteristics in the urgent, semi-urgent, and delayed cholecystectomy groups.

	Urgent group (n = 57)	Semi-urgent group (n = 44)	Delayed group (n = 7)	*P* value
Sex, M/F	29/28	27/17	1/6	.162
**Mean age, years (range)**	80 (35–95)	73 (26–94)	79 (63–83)	**.020**
BMI, kg/cm^2^ (range)	22.9 (15.8–35.4)	24.3 (17.5–35.0)	24.0 (21.4–31.2)	.095
ASA-PS ≥ 3 (%)	15 (26.3%)	10 (22.7%)	0 (%)	.302
Preoperative biliary drainage (%)	52 (91.2%)	38 (86.3%)	7 (100%)	.462
**Murphy's sign (%)**	37 (64.9%)	22 (50.0%)	1 (14.3%)	**.022**
**WBC/μL (range)**	12600 (4120–30700)	10690 (2000–27030)	6480 (4900–7970)	**.012**
**CRP, mg/μL (range)**	5.4 (0.0–31.0)	9.9 (0.1–32.3)	0.1 (0–1.0)	**.003**
**Acute cholecystitis G1/2/3**	32/18/7	10/31/3	6/1/0	**.001**
Computed tomography findings				
Wall thickness	50 (87.7%)	35 (79.5%)	6 (85.7%)	.613
**Gallstone**	39 (68.4%)	34 (77.2%)	1 (14.3%)	**.004**
Enhanced surrounding tissue	39 (68.4%)	31 (70.5%)	7 (100%)	.294
Effusion around gallbladder	9 (15.8%)	13 (29.5%)	2 (28.6%)	.190
Ascites	6 (10.5%)	3 (6.8%)	0 (0%)	.659
Liver abscess	3 (5.3%)	3 (6.8%)	1 (14.3%)	.812
**Abscess around gallbladder**	0 (0%)	6 (13.6%)	0 (0%)	.**010**
Diabetes mellitus (%)	20 (35.1%)	9 (20.4%)	2 (%)	.321
Hypertension (%)	29 (50.9%)	20 (45.4%)	6 (85.7%)	.165
Pulmonary disease (%)	9 (15.8%)	3 (6.8%)	0 (0%)	.240
Cardiovascular disease (%)	12 (21.1%)	13 (29.5%)	1 (14.3%)	.583
Chronic kidney disease (%)	6 (10.5%)	6 (13.6%)	2 (28.6%)	.417

Variables in bold are statistically significant (*p* < 0.05). ASA, American Society of Anesthesiologists; BMI, body mass index; CRP, C-reactive protein; F, female; G, grade; M, male; PS, physical status; WBC, white blood cell.

### 3.4 Comparison of perioperative outcomes in urgent, semi-urgent, and delayed cholecystectomy groups

The patients’ intra- and postoperative outcomes are shown in Table 4. The operative procedures were the same among the three groups (*p* = 0.705). There were no significant differences in operation time (*p* = 0.548), intraoperative bleeding (*p* = 0.106), postoperative complications (*p* = 0.686), or postoperative hospital stay (*p* = 0.475).

**Table 4:** Surgical outcomes of the urgent, semi-urgent, and delayed operation groups.

	Urgent group (n = 57)	Semi-urgent group (n = 44)	Delayed group (n = 7)	*P* value
Operative procedure				.705
LC (%)	46 (80.7%)	33 (75.0%)	6 (85.7%)	
OC (%)	11 (19.3%)	11 (25.0%)	1 (14.3%)	
Operation time, min (range)	135 (87–222)	150 (71–253)	130 (112–242)	.548
Intraoperative bleeding, mL (range)	35 (0–500)	60 (0–1200)	0 (0–100)	.106
Postoperative complications ≥ CD IIIa, n (%)	8 (14.0%)	6 (13.6%)	0 (0%)	.686
Postoperative stay, days (range)	9 (4–41)	9 (4–86)	5 (3–14)	.475

Variables in bold are statistically significant (*p* < 0.05). Continuous variables are expressed as median (range). CD, Clavien-Dindo classification; LC, laparoscopic cholecystectomy; OC, open cholecystectomy.

## 4 Discussion

To our knowledge, this is the first study to validate the efficacy of early cholecystectomy in patients with comorbid acute cholangitis and acute cholecystitis per the TG13 guidelines [1]. Early cholecystectomy performed within 72 h after symptom onset is recommended by the TG13; however, recent data show that this time limitation could be lengthened from 72 h to 7 days without increasing perioperative complications [17,20]. Our study demonstrated that patients with concomitant biliary infections could be good candidates for early cholecystectomy from equivalent postoperative outcomes. Early cholecystectomy would be optimal surgical timing once the acute cholangitis had subsided. Moreover, our study revealed that postoperative outcomes were feasible and safe between patients with concomitant acute cholangitis and acute cholecystitis; additionally, optimal surgical timing is acceptable within 14 days after recovery from acute cholangitis. Our strategy for managing concomitant biliary infections is to perform early cholecystectomy when the patient's general condition stabilizes and they can tolerate general anesthesia. Acute cholecystitis easily recurs, and repeated bouts of inflammation would inhibit safe cholecystectomy. Conservative treatment failure for acute cholecystitis could indicate a more severe general condition than that at admission. Our aggressive surgical approach could be beneficial for preventing biliary infection recurrence and reducing the incidence of intraoperative biliary injury.

The benefit of early cholecystectomy after acute cholangitis treatment is preventing acute cholecystitis recurrence during the long waiting period [2,7]. Mizrahi et al. showed that the best surgical management consists of cholecystectomy within 6 weeks after symptom onset rather than beyond 7 weeks of waiting time after percutaneous cholecystostomy for acute cholecystitis [21]. In line with previous reports, our data support the superiority of early cholecystectomy because neither postoperative complications nor intraoperative biliary injury differed between delayed or semi-urgent cholecystectomy. In clinical situations, the first-line approach for concurrent acute cholangitis with acute cholecystitis is to provide urgent biliary drainage or antibiotic therapy according to acute cholangitis severity grade because acute cholangitis frequently progresses to septic shock or organ disorders. In the acute inflammation period around 7 days after an acute cholecystitis attack, the tissue surrounding the gallbladder is frequently edematous rather than fibrotic. Waiting for intervention allows maturation of the surrounding inflammation between the gallbladder and the hepatic bed, which makes it more difficult to dissect this layer due to the complete fibrotic changes [22]. The fibrotic tissue between the gallbladder serosa structured by chronic rather than acute inflammation would obscure Calot's triangle. The hypothesis of the “cool-down” effect used to create the interval from acute inflammation to delayed surgical intervention would not be obtained due to high rates of biliary complications [[21], [22], [23]]. The “cool-down” effect is achieved only in patients without previous biliary complications, and repeated bouts of acute cholecystitis can create severe adhesions even if an adequate interval period is preserved.

Based on the advantages of early cholecystectomy for preventing major bile duct injury and acute cholecystitis recurrence, early cholecystectomy should be considered after the successful treatment of patients with comorbid acute cholecystitis and acute cholangitis [7,24]. In our institute, prior to surgery, the extra-biliary anatomy is evaluated to facilitate safe dissection during cholangiography or MRCP. Among the various biliary drainage types, ENBD is used in our institute because successful drainage can be confirmed by checking the amount of bile juice during the perioperative period. Consistent with the findings of a previous study, cholangiography by ENBD is a useful and safe tool for preventing iatrogenic bile duct injury [25]. Intraoperative cholangiography by ENBD can be performed in severe inflammation cases in which dissecting Calot's triangle is difficult. Moreover, based on our results, upfront treatment for acute cholangitis is essential for achieving low postoperative mortality. Endo et al. reported that jaundice was the independent predictive factor for 30-day mortality in G1 and G2 acute cholecystitis [26].

Postoperative complications occurred in 14% of patients in the concomitant acute cholangitis group and 11% of patients in the non-acute cholangitis group, which is on the lower side of values reported in the literature [2,3,20,22]. Biliary leakage is one of the most concerning postoperative complications. Choosing the appropriate surgical procedure is important in acute abdominal surgery. In cases of severe local inflammation and a fragile cystic duct, an open procedure and bailout surgery should be used to accurately localize Calot's triangle and avoid biliary injury.

This study has some limitations. First, this was a retrospective single-center study with a relatively small sample size. Moreover, patients with severe acute cholangitis were excluded due to the presence of other severe conditions and resulting poor surgical candidacy. It is very likely that future multi-center studies will examine the effectiveness of urgent cholecystectomy on the clinical outcomes of such patients.

## 5 Conclusions

This study demonstrated the feasibility and safety of cholecystectomy performed within 14 days after symptom onset for patients with comorbid acute cholecystitis and acute cholangitis after successful treatment for acute cholangitis.

## Provenance and peer review

Not commissioned, externally peer reviewed.

## Ethical approval

This work has been reported in line with the Strengthening the Reporting of Cohort Studies in Surgery criteria.

## Funding

There was no source of funding for my research.

## Author contribution

**Tomoyuki Abe**, **Keiji Hanada**, **Tomoyuki Minami** and **Hironobu Amano** wrote the manuscript. The remaining authors contributed to the collection, analysis, and interpretation of data. Tomoaki Bekki, Tomoyuki Abe, Hironobu Amano, Masahiro Nakahara, and Toshio Noriyuki performed the surgery. Shuji Yonehara performed the pathological diagnosis of the disease. All authors conceived the study, participated in its design and coordination, and helped to draft the manuscript. All authors have read and approved the final manuscript.

## Conflicts of interest

The authors declare no conflicts of interest.

## Research registration unique identifying number (UIN)

researchregistry4056.

## Guarantor

Our manuscript was guaranteed by all authors.

## Appendix A Supplementary data

Multimedia component 1 Multimedia component 1

Data_profile Data_profile
